# Alleviation of drought stress by melatonin foliar treatment on two flax varieties under sandy soil

**DOI:** 10.1007/s12298-020-00789-z

**Published:** 2020-04-18

**Authors:** Mervat Shamoon Sadak, Bakry Ahmed Bakry

**Affiliations:** 1 Botany Department, Agricultural and Biological Division, National Research Centre, Dokki, Giza, Egypt; 2 Field Crop Department, Agricultural and Biological Division, National Research Centre, 33 El Bohouth St., P.O. 12622, Dokki, Giza, Egypt

**Keywords:** Antioxidant enzymes, Drought tolerance, Flax, Free amino acids, IAA, Melatonin, Phenolics and yield

## Abstract

The role of melatonin treatments on improving plant tolerance against drought stress is clear, while its special role and influences are poorly investigated. Thus, the effect of external treatment with different concentrations (2.5, 5.0 and 7.5 mM) of melatonin on two varieties of flax plant (Letwania-9 and Sakha-2) growth, some biochemical aspects and yield under normal [100% water irrigation requirements (WIR)] and drought stress conditions (75% and 50% WIR) in sandy soil were investigated in this study. Drought stress decreased significantly different growth parameters, photosynthetic pigments, yield and yield components of the two studied flax varieties. While, it increased significantly phenolic contents, total soluble sugars (TSS), proline and free amino acids as well as some antioxidant enzymes (superoxide dismutase, catalase, peroxidase and polyphenol oxidase). Meanwhile, external treatment of melatonin (2.5, 5.0 and 7.5 mM) increased significantly different growth and yield parameters as well as the studied biochemical and physiological aspects under 100% WIR. Also, melatonin treatment could alleviate the adverse effects of drought stress and increased significantly growth parameters, yield and quality of the two varieties of flax plant via improving photosynthetic pigments, indole acetic acid, phenolic, TSS, proline free amino acids contents and antioxidant enzyme systems, as compared with their corresponding untreated controls. Foliar treatment of 5.0 mM melatonin showed the greatest growth, the studied biochemical aspects and yield quantity and quality of Letwania-9 and Sakha-2 varieties of flax plants either at normal irrigation or under stress conditions. Finally we can conclude that, melatonin treatment improved and alleviated the reduced effect of drought stress on growth and yield of two flax varieties through enhancing photosynthetic pigment, osmoptrotectants and antioxidant enzyme systems. 5 mM was the most effective concentration.

## Introduction

Flax plant (*Linum usitatissimum* L.), one of the most important crops grown in Egypt, is used as seed, fiber and dual purpose plant (fibers and seeds). Flax seeds contain 30–40 percent of edible oil with high nutritional value resulting from the high amount of essential fatty acids (linoleic acid, linolenic acid and oleic acid) as well as, proteins, mucilage and cyanogenic glycosides. In Egypt, flax is considered second fiber crop after cotton. This plant used in production of feeding stuff for poultry and animals, as well as, different types of compact wood (particle board) (Bakry et al. 2013). Various flax varieties greatly differ in yield and yield components (Darja and Trdan 2008).

Drought stress (as an environmental stress) is severe deficiency of water which depress plant growth, development and productivity especially in arid and semiarid regions (Battipaglia et al. 2014). The increase in aridity is expected due to the increase in global climate changes in various regions all over the world (Blum 2017). Drought stress affect adversely plant growth, photosynthetic pigments, water and nitrogen use efficiency alterations, changes in cell structure and activities of key enzymes in various plant species (He et al. 2016; Chen et al. 2019). Also, drought stress caused oxidative damage to plant cells via increasing accumulation of reactive oxygen species (ROS) which reduce photosynthesis, stomatal closure and alter the activities of enzymes. ROS formation is considered a threat to cell as it causes electron leakage, lipid peroxidation and subsequent membrane damage, as well as damage to nucleic acids and proteins (Maksup et al. 2014). To decrease these damages, plants have evolved different pathways such as increasing antioxidant compounds either non enzymatic antioxidant (as glutathione, ascorbic acid carotenoids, α-tocopherols) or enzymatic antioxidants (including superoxide dismutase (SOD), ascorbate peroxidase (APX), catalase (CAT) and guaiacol peroxidase (GPX) (Abd Elhamid et al. 2014). Another antioxidants compound which improves plant tolerance in plant tissue is different phenolic compounds. Phenolic compounds are potential antioxidants acting as ROS-scavenging compounds (Rice-Evans et al. 1997). Thus, more studies are needed on plant response to drought stress (Petit et al. 1999). Recently, use of efficient, economic and inexpensive compounds for improving and enhancing plant tolerance to biotic and abiotic stress such as drought stress has been reported. One of these compounds is melatonin.

Melatonin is a new plant growth regulator efficient in enhancing environmental stress tolerance of different crops. Melatonin is present in various living organisms (Tan et al. 2012) with various levels in plant (Arnao and Hernández-Ruiz 2014; Fleta-Soriano et al. 2017; Alam et al. 2018). The lipophilic and hydrophilic nature of melatonin gives it the possibility of passing through morpho-physiological barriers easily resulting in rapid transport of the molecule into plant cells (Tan et al. 2012). Melatonin plays many important roles in vegetative growth improvement, rooting and flowering (Arnao and Hernández-Ruiz 2014; Hardeland 2015). Also, melatonin could enhance plant tolerance of multiple stresses as well as helps in homeostasis of various ions (Arnao and Hernández-Ruiz 2015; Wei et al. 2015; Li et al. 2016, 2018, 2019). Melatonin is a well-documented antioxidant in various crops (Zhang and Zhang 2014). Improving antioxidant abilities of plant is a general effective role of melatonin, thus causing increase in plant stress tolerance (Arnao and Hernández-Ruiz 2015; Zhang et al. 2015). Exogenous treatment of melatonin has been found to increase stress tolerance of plant (Zuo et al. 2017; Sun et al. 2018). Even though, many investigations have stated that melatonin external treatment can improve drought tolerance, its specific role and the underlying mechanism of melatonin’s role on plant drought tolerance are poorly understood. Firstly, the effect of melatonin on plant drought tolerance has been studied in only a few plant species, and only a quite small number of these investigations have focused on highly important crops. Secondly, these investigations have added melatonin by either adding it into the soil or into a nutrient solution, both of which are inconvenient in field crop production. Third, the majority of these investigations have been done under environmentally controlled conditions, such as in growth chambers or greenhouses, thus their results cannot accurately reflect the performance of melatonin with respect to stress tolerance in the field environment (Li et al. 2018). Therefore, the performance and mechanism of melatonin’s effect on drought tolerance needs further study, especially in highly important crops under field environmental conditions.

So, in this investigation, our aim was to study the enhancing role of foliar treatment of melatonin on growth and yield of two varieties of flax plant grown under drought stress in sandy soil.

## Materials and methods

Two field experiments were carried out at the experimental station of National Research Centre, Al Nubaria district El-Behira Governorate-Egypt, in 2015/2016 and 2016/2017 winter seasons. Soil of the both experimental sites was sandy soil. Mechanical, chemical and nutritional analysis of the experimental soils is reported in Table 1 according to Chapman and Pratt (1978).

**Table 1:** Some physical and chemical characteristics of the experimental soil

Sand (%)	Clay (%)	Silt (%)	pH	Organic matter (%)	CaCO_3_ (%)	E.C. (dS/m)	Soluble N (ppm)	Available P (ppm)	Exchangeable K (ppm)
85.3	4.0	10.7	7.84	0.4	1.0	3.95	8.1	3.2	20

The experimental design was split-split plot design, using three replicates where water irrigation requirements (100%, 75% and 50%) occupied the main plots, two flax cultivars (Letwania-9 and Sakha-2) were allocated in sub plots and the concentrations of melatonin (0.0, 2.5 mM, 5 mM and 7.5 mM) were allocated at random in sub–sub plots. Flax seeds of Letwania-9 and Sakha-2 cultivars were sown on 17th November in the two winter seasons in rows 3.5 meters long, and the distance between rows was 20 cm apart, plot area was 10.5 m^2^ (3.0 m in width and 3.5 m in length). The seeding rate was 2000 seeds/m^2^. Pre-sowing, 150 kg/fed of calcium super-phosphate (15.5% P_2_O_5_) were used. Nitrogen was applied after emergence in the form of ammonium nitrate 33.5% at rate of 75 kg/fed in five equal doses. Potassium sulfate (48% K_2_O) was added at two equal doses of 50 kg/fed. Irrigation was carried out using the new sprinkler irrigation system where water was added every 7 days as per schedule in Table 2 for water requirements/fed.

### Irrigation water requirements

Three irrigation water requirements was calculated using Penman–Monteith equation and crop coefficient according to Allen et al. (1989). The average amount of irrigation water applied with sprinkler irrigation system were 2500, 1875 and 1250 m^3^ fed.^−1^ season^−1^ as (100%, 75% and 50%, respectively) for both seasons of in 2015/2016 and 2016/2017.

The amounts of irrigation water were calculated according to the following equation:

$${\text{IWR}} = \left( {\frac{\text{ETo*Kc*Kr*I}}{\text{Ea}} + {\text{LR}}} \right) *4.2$$

where IWR = irrigation water requirement m^3^/fed/irrigation, ETo = reference Evapotranspiration (mm/day), Kc = crop coefficient, Kr = reduction factor (Keller and Karmeli 1975), I = irrigation interval, day, Ea = irrigation efficiency, 90%, LR = leaching requirement = 10% of the total water amount delivered to the treatment.

Foliar application of different concentrations of melatonin (0.0, 2.5 mM, 5 mM and 7.5 mMl) were carried out twice at rate of (200 L/fed); where plants were sprayed after 30 and 45 days from sowing. Plant samples were taken after 60 days from sowing for measurements of growth characters and some biochemical parameters. Growth parameters were in terms of, shoot length (cm), shoot fresh and dry weight (g), roots length (cm), root fresh and dry weight (g). Chemical analysis measured were photosynthetic pigments, total phenol contents and some antioxidant enzymes such as polyphenol oxidase (PPO), peroxidase (POX), catalase (CAT) and superoxide dismutase (SOD). Plant samples were dried in an electric oven with drift fan at 70 °C for 48 h till constant dry weight for determination of total soluble sugars (TSS), free amino acids and proline contents. Flax plants were pulled when signs of full maturity were appeared, then left on ground to suitable complete drying. Capsules were removed carefully. At harvest, plant height (cm), fruiting zone length (cm), number of fruiting branches/plant, number of capsules/plant, seed yield/plant (g), biological yield/plant (g) and 1000 seeds wt (g), were recorded on random samples of ten guarded plants in each plot. Also, seed yield/fed (kg/Fed), straw yield (kg/fed), biological yield (kg/fed) and oil yield (kg/Fed) were studied.

*Chemical analysis:* Photosynthetic pigments contents (chlorophyll a and b and carotenoids) in fresh leaves were estimated using the method of Lichtenthaler and Buschmann (2001). Total phenol content was measured as described by Danil and George (1972). Total soluble sugars (TSS) were extracted by the method of Homme et al. (1992) and analyzed using Spekol SpectrocololourimeterVEB Carl Zeiss (Yemm and Willis,^1954^). Free amino acids were extracted according to Vartanian et al. (1992) and estimated according to (Yemm and Cocking 1955). Proline was extracted as free amino acid and assayed according to Bates et al. (1973). The method used for extracting the enzyme is that of MuKherjee and Choudhuri (1983). Polyphenol oxidase (PPO, EC 1.10.3.1) activity assayed using the method of Kar and Mishra (1976). Peroxidase (POX, EC 1.11.1.7) activity assayed using the method of Bergmeyer (1974). Catalase (CAT, EC 1.11.1.6) activity was assayed according to the method of Chen et al. (2000). Superoxide dismutase (SOD, EC 1.12.1.1) activity was measured according to the method of Dhindsa et al. (1981). The enzyme activities were calculated by Kong et al. (1999). Seed oil content was determined using Soxhlet apparatus and petroleum ether (40–60 °C) according to AOAC (1990).

### Statistical analysis

The data were statistically analyzed on complete randomized design under split–split plot system according to Snedecor and Cochran (1980). since the trend was similar in both seasons, the homogeneity test Bartlet’s equation was applied and the combined analysis of the two seasons was done according to the method of Gomez and Gomez (1984). Means were compared by using least significant difference (LSD) at 5%.

## Results

### Growth parameters

The presented data in Table 2 shows the effect of foliar treatment of two flax varieties with different concentrations of melatonin (0.0 mM, 2.5 mM, 5 mM and 7.5 mM) grown under different water irrigation requirements WIR (100%, 75% and 50%) on growth parameters. Drought stress (75% and 50% WIR) decreased gradually and significantly shoot length, fresh and dry weight, while increased significantly and gradually root length, fresh and dry weight of root relative to those plants irrigated with 100% WIR (control plants) of the two varieties. It is clear that, Letwania-9 variety was more tolerant to drought stress in relation to Sakha-2 variety under the two drought stress levels (75% and 50%). 75% irrigation water requirement caused 10.48%, 8.90% and 30.71% decrease in Letwania-9 variety, while the percent of decreases were 15.54%, 16.53% and 24.18% in Sakha-2 variety of shoot length, fresh and dry weight, respectively as compared with plants irrigated with 100% irrigation water requirement. On the other hand, foliar treatment of the two tested varieties of flax plants with different concentrations of melatonin (2.5, 5.0 and 7.5 mM) increased the above mentioned growth parameters (shoot length, fresh and dry weight), as well as it caused more increases in root length, fresh and dry weight of root relative to their untreated controls under different WIR either at normal WIR (100%) or drought stressed WIR (75% and 50%). 5 mM melatonin foliar treatment was the most effective concentration over the other two concentrations (2.5 and 7.5 mM) as it caused the highest increases in most studied parameters (Table 2).

**Table 2:** Effect of melatonin (0.0, 2.5, 5 and 7.5 mM) on growth parameters of two flax varieties under different water irrigation requirements (combined data of two seasons)

Varieties	WIR	Melatonin (mM)	Plant height (cm)	Shoot fresh wt. (g)	Shoot dry wt. (g)	Root length (cm)	Root fresh wt. (g)	Root dry wt. (g)
Letwania-9	100	0	63 ± 0.33	3.37 ± 0.33	1.40 ± 0.09	7 ± 0.33	0.53 ± 0.06	0.31 ± 0.01
		2.5	74 ± 4.93	7.03 ± 0.33	2.08 ± 0.14	9 ± 0.58	1.44 ± 0.14	0.35 ± 0.03
		5.0	78 ± 2.67	9.43 ± 1.20	3.26 ± 0.24	10 ± 0.33	1.59 ± 0.08	0.37 ± 0.04
		7.5	70 ± 1.33	7.27 ± 0.58	2.84 ± 0.28	8 ± 0.58	1.43 ± 0.08	0.33 ± 0.00
	75	0	57 ± 1.00	3.07 ± 0.50	0.97 ± 0.03	11 ± 0.57	0.79 ± 0.12	0.40 ± 0.03
		2.5	70 ± 3.00	5.53 ± 0.61	1.61 ± 0.22	16 ± 0.57	1.45 ± 0.02	0.48 ± 0.00
		5.0	74 ± 1.15	6.93 ± 0.09	2.08 ± 0.24	16 ± 0.33	1.58 ± 0.12	0.52 ± 0.00
		7.5	73 ± 1.53	4.23 ± 0.71	1.78 ± 0.36	15 ± 0.33	1.78 ± 0.12	0.47 ± 0.02
	50	0	54 ± 1.53	2.77 ± 0.49	0.80 ± 0.09	12 ± 0.58	1.29 ± 0.14	0.48 ± 0.01
		2.5	61 ± 0.88	3.67 ± 0.19	1.25 ± 0.09	13 ± 0.33	1.41 ± 0.07	0.45 ± 0.01
		5.0	67 ± 2.03	5.60 ± 0.10	1.94 ± 0.18	15 ± 0.58	1.77 ± 0.11	0.50 ± 0.01
		7.5	63 ± 2.40	3.33 ± 0.41	1.55 ± 0.14	16 ± 0.58	1.87 ± 0.06	0.49 ± 0.00
Sakha-2	100	0	64 ± 0.88	2.60 ± 0.06	0.91 ± 0.04	7 ± 0.58	0.53 ± 0.04	0.18 ± 0.02
		2.5	71 ± 2.03	6.63 ± 0.41	1.86 ± 0.17	9 ± 0.33	1.37 ± 0.19	0.24 ± 0.01
		5.0	80 ± 0.58	7.33 ± 0.55	2.22 ± 0.13	12 ± 0.33	2.00 ± 0.15	0.26 ± 0.01
		7.5	69 ± 2.19	4.97 ± 0.12	1.62 ± 0.27	11 ± 0.58	1.96 ± 0.03	1.97 ± 0.05
	75	0	54 ± 1.86	2.17 ± 0.11	0.69 ± 0.01	12 ± 0.33	0.62 ± 0.03	0.15 ± 0.02
		2.5	68 ± 0.88	3.33 ± 0.07	1.38 ± 0.02	12 ± 0.33	0.77 ± 0.06	0.22 ± 0.01
		5.0	74 ± 3.06	4.60 ± 0.00	1.68 ± 0.85	14 ± 0.33	0.99 ± 0.07	0.26 ± 0.01
		7.5	70 ± 2.65	3.20 ± 0.26	1.35 ± 0.01	15 ± 1.00	1.46 ± 0.16	0.31 ± 0.00
	50	0	44 ± 1.76	1.60 ± 0.07	0.44 ± 0.03	15 ± 0.33	1.01 ± 0.07	0.15 ± 0.01
		2.5	61 ± 1.53	2.43 ± 0.03	0.85 ± 0.05	15 ± 0.33	2.16 ± 0.33	1.66 ± 0.07
		5.0	65 ± 1.86	3.57 ± 0.15	0.99 ± 0.02	15 ± 0.33	3.96 ± 0.15	0.31 ± 0.03
		7.5	61 ± 1.76	3.07 ± 2.33	0.88 ± 0.02	15 ± 0.33	3.53 ± 0.21	0.27 ± 0.15
LSD_0.05_			3.13	0.45	0.35	1.10	0.28	0.05

Each value represents the mean of three replicates ± SE

### Photosynthetic pigments

**Fig. 1:**
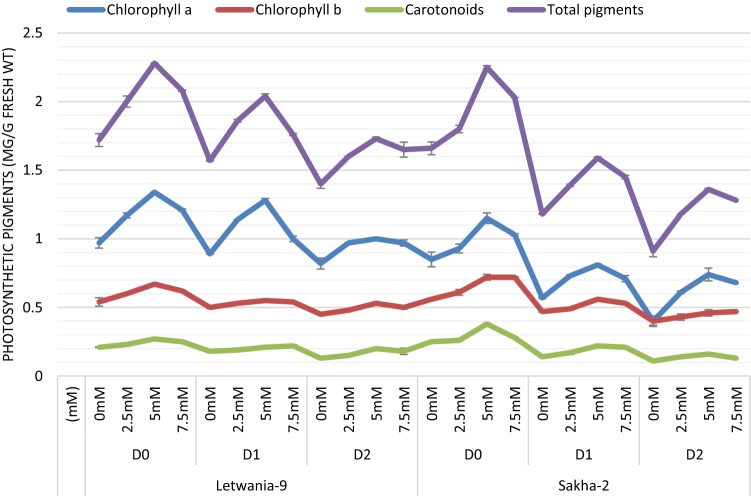
Effect of melatonin on photosynthetic pigments (mg/g Fwt) of two flax varieties under different water irrigation requirements (combined data of two seasons) LSD at 5%, chlorophyll a 0.11, chlorophyll b 0.07, carotenoids 0.03 and total pigments 0.17). Each value represents the mean of three replicates ± SE

Irrigation of two varieties (Letwania-9 and Sakha-2) of flax plants with low water irrigation requirements (75% and 50%) caused significant and gradual decreases in all components of photosynthetic pigments (chlorophylls a, b and carotenoids and consequently total photosynthetic pigments) relative to the control plants which were irrigated with 100% WIR (Fig. 1). On the other hand, melatonin foliar treatment with different concentrations (2.5, 5.0 and 7.5 mM) improved photosynthetic pigments of the two flax varieties under normal and stressed conditions compared with those untreated plants. 5.0 mM was the most effective treatment as it caused the highest increases in all photosynthetic pigments components of the two varieties of flax plant under different water irrigation requirement.

### Changes in phenolics

**Fig. 2:**
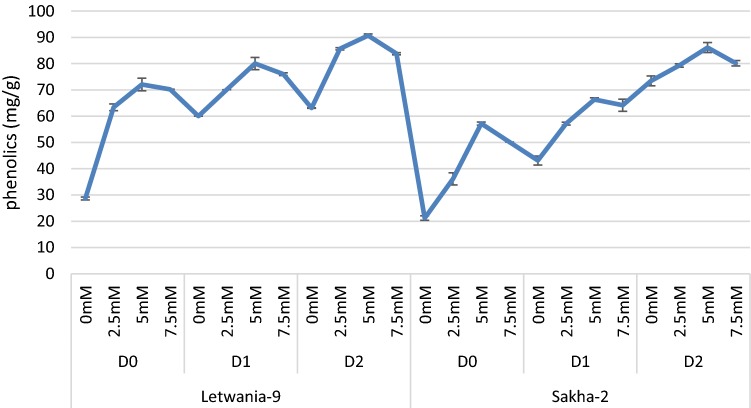
Effect of melatonin on phenolic (mg/g wt) of two flax varieties under different water irrigation requirements (combined data of two seasons) (LSD at 5% 3.10). Each value represents the mean of three replicates ± SE

Subjecting flax plant (Letwania-9 and Sakha-2 varieties) to different water irrigation requirements WIR 75% and 50% caused significant and gradual increases in phenolic contents of the two varieties of flax plant relative to their controls plant (100%) (Fig. 2). Whereas, melatonin foliar treatment with different concentrations (2.5, 5.0 and 7.5 mM) caused gradual increases in phenolics contents in the two varieties of flax plant as compared with their corresponding untreated controls (Fig. 2). It is clear that 5.0 mM was the most effective concentration as it caused the highest increases in phenolics under different WIR of the tested flax varieties (Letwania-9 and Sakha-2).

### Changes in some osmorotectants

The changes in some osmoprotectants as total soluble sugars TSS %, proline and free amino acids contents of two varieties Letwania-9 and Sakha-2 of flax plants in response to foliar treatment of different concentrations (2.5, 5.0 and 7.5 mM) of melatonin under different water irrigation requirements 100%, 75% and 50% are presented in Table 3. Decreased WIR 75% and 50% increased gradually and markedly TSS, proline and free amino acids contents in flax two varieties as compared with plants with 100% WIR. Moreover, different melatonin concentrations (2.5, 5.0 and 7.5 mM) caused marked increases in the studied osmoprotectants (TSS, proline and free amino acids) of the two studied varieties as compared with their corresponding untreated controls under normal irrigation conditions (100%) or stressed conditions (75% and 50%). 5.0 mM was the most effective treatment on increasing different osmoprotectants contents of flax plant varieties (Table 3).

**Table 3:** Effect of melatonin on phenolic, total soluble sugars (TSS  %), proline and free amino acids contents (mg/g wt) of two flax varieties under different water irrigation requirements (combined data of two seasons)

Varieties	Drought stress (%)	Melatonin (mM)	TSS	Proline	Free amino acids
Letwania-9	100	0	4.14 ± 0.05	28.00 ± 1.55	233.9 ± 15.59
		2.5	4.25 ± 0.04	30.00 ± 0.32	259.7 ± 3.20
		5.0	4.93 ± 0.31	30.85 ± 0.15	229.9 ± 0.29
		7.5	4.61 ± 0.09	30.85 ± 0.29	311.6 ± 2.89
	75	0	5.98 ± 0.21	40.72 ± 0.44	323.9 ± 6.35
		2.5	6.18 ± 0.09	41.12 ± 0.34	339.6 ± 0.88
		5.0	6.77 ± 0.03	46.05 ± 0.10	390.9 ± 0.46
		7.5	6.27 ± 0.07	41.62 ± 1.45	363.3 ± 3.47
	50	0	5.79 ± 0.04	48.18 ± 3.43	500.6 ± 10.26
		2.5	6.94 ± 0.04	48.78 ± 0.88	503.0 ± 11.48
		5.0	7.93 ± 0.11	52.25 ± 0.87	546.3 ± 0.83
		7.5	6.43 ± 0.22	50.78 ± 0.07	522.9 ± 1.87
Sakha-2	100	0	5.45 ± 0.06	15.65 ± 0.54	178.8 ± 5.65
		2.5	5.88 ± 0.08	17.95 ± 1.05	200.4 ± 0.76
		5.0	7.77 ± 0.26	22.45 ± 0.25	246.9 ± 9.93
		7.5	7.59 ± 0.41	20.31 ± 0.55	225.4 ± 0.61
	75	0	7.07 ± 0.19	24.84 ± 0.31	304.7 ± 3.19
		2.5	8.92 ± 0.23	29.05 ± 0.36	313.2 ± 5.83
		5.0	9.31 ± 0.35	31.47 ± 0.58	334.3 ± 3.89
		7.5	7.94 ± 0.35	29.84 ± 0.46	321.7 ± 6.75
	50	0	7.58 ± 0.31	40.12 ± 0.63	422.4 ± 3.10
		2.5	8.96 ± 0.12	40.45 ± 0.49	428.3 ± 3.67
		5.0	9.76 ± 0.33	43.12 ± 0.12	464.0 ± 3.82
		7.5	8.40 ± 0.19	41.31 ± 0.35	430.9 ± 6.87
LSD at 5%			0.77	1.67	33.17

Each value represents the mean of three replicates ± SE

### Changes in antioxidant enzyme activities

**Fig. 3:**
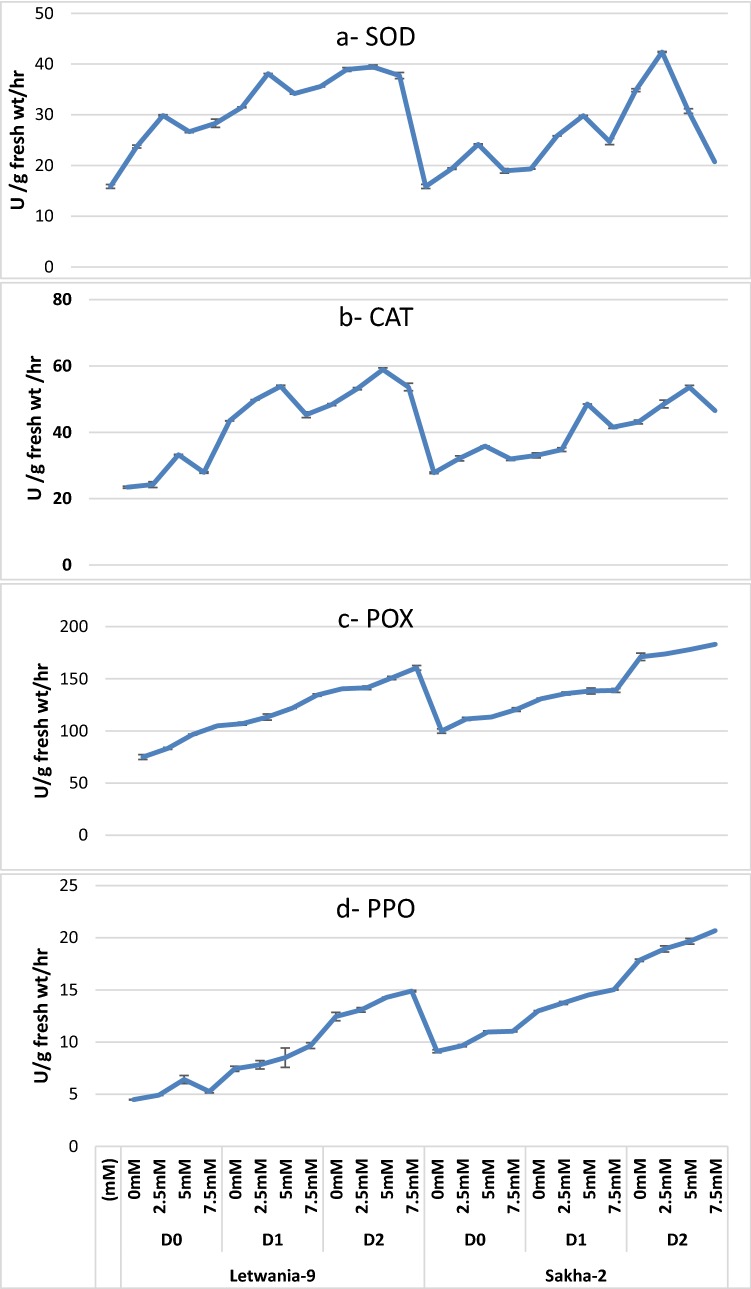
Effect of melatonin on antioxidant activities (SOD, CAT, POX and PPO) (U activity/g fresh wt/h) of two flax varieties under different water irrigation requirements (combined data of two seasons) LSD at 5%: SOD 1.45, CAT 1.25, POX, 7.75 and PPO 1.61). Each value represents the mean of three replicates ± SE

The antioxidant enzymes data presented in Fig. 3a–d shows that exposure of the two varieties of flax plant to drought stress (by decreasing water irrigation requirements to 75% and 50%) increased significantly the activities of the tested enzymes as superoxide dismutase (Fig. 3a, SOD), catalase (Fig. 3b, CAT), peroxidase (Fig. 3c, POX) and polyphenol oxidase (Fig. 3d, PPO) as compared with those plants irrigated with 100% WIR (control plant). Moreover, different concentrations of melatonin (2.5, 5.0 and 7.5 mM) caused more significant increases in different studied enzymes (Fig. 3a–d) as compared with untreated control plants under their corresponding WIR (100%, 75% and 50%). The highest different enzyme activities were obtained with foliar treatments with 5.0 mM melatonin under different WIR on the two tested varieties Letwania-9 and Sakha-2 compared with the other two concentrations (2.5 and 7.5 mM) of melatonin.

### Yield attributes

Data in Table 4a and b shows that, yield and yield attributes (plant height, biological yield/plant, fruiting zone length, number of fruiting branches and capsules/plant, 1000 seeds wt, seed yield/fed, biological yield/fed and straw yield) of flax two varieties (Letwania-9 and Sakha-2) were decreased gradually and markedly by lowering water irrigation requirements (75% and 50%) as compared to control plants (100%). On the other hand, foliar treatment of the two flax varieties with different concentrations of melatonin (2.5, 5.0 and 7.5 mM) caused significant increases in all parameters of yield components under normal irrigation requirements (100), as well as under reduced water irrigation requirements (75% and 50%). Data show the superiority of Letwania-9 variety over Sakha-2 in yield and yield components.

**Table 4:** (a and b) Effect of melatonin on yield and yield attributes of two flax varieties grown under different water irrigation requirements (combined data of two seasons)

Varieties	Drought stress (%)	Melatonin (mM)	Technical stem length	Plant height (cm)	Biological yield/plant (g)	Fruiting zone length (cm)	No. of fruiting branches/plant	No. of capsules/plant	1000 Seeds wt (g)
Letwania-9	100	0	95 ± 0.67	83 ± 0.88	2.46 ± 0.46	12 ± 0.58	8.0 ± 0.58	17.7 ± 0.33	5.95 ± 0.09
		2.5	102 ± 1.00	87 ± 1.45	2.84 ± 0.37	15 ± 0.67	8.3 ± 0.58	23.0 ± 0.00	6.57 ± 0.09
		5.0	112 ± 3.18	92 ± 3.28	3.37 ± 0.17	20 ± 0.58	12.0 ± 0.58	27.3 ± 1.20	6.66 ± 0.14
		7.5	103 ± 1.53	90 ± 1.15	2.92 ± 0.36	13 ± 0.58	10.7 ± 0.88	20.3 ± 0.33	5.95 ± 0.10
	± 75	0	81 ± 0.33	72 ± 0.67	2.28 ± 0.27	9 ± 0.58	6.3 ± 0.33	15.7 ± 0.67	4.68 ± 0.05
		2.5	87 ± 0.88	76 ± 0.33	2.28 ± 0.14	11 ± 1.20	8.0 ± 0.58	17.7 ± 0.67	5.14 ± 0.10
		5.0	100 ± 0.58	87 ± 0.33	2.71 ± 0.23	13 ± 0.33	12.0 ± 0.58	24.7 ± 2.18	5.74 ± 0.12
		7.5	96 ± 0.88	83 ± 1.00	2.53 ± 0.10	13 ± 0.20	10.7 ± 0.67	19.7 ± 0.88	5.21 ± 0.07
	50	0	71 ± 1.20	64 ± 1.00	1.72 ± 0.01	7 ± 0.33	3.7 ± 0.33	11.0 ± 0.33	3.84 ± 0.19
		2.5	80 ± 0.33	71 ± 0.67	1.89 ± 0.02	9 ± 0.33	4.0 ± 0.33	13.3 ± 0.57	4.04 ± 0.01
		5.0	92 ± 2.51	84 ± 2.96	1.98 ± 0.07	9 ± 0.67	5.0 ± 0.88	16.0 ± 0.57	4.28 ± 0.18
		7.5	83 ± 0.58	73 ± 0.88	1.68 ± 0.02	10 ± 0.67	7.3 ± 0.00	12.3 ± 0.33	4.10 ± 0.18
Sakha-2	100	0	79 ± 1.76	65 ± 2.52	1.72 ± 0.46	14 ± 0.88	7.0 ± 0.58	17.7 ± 0.33	5.02 ± 0.13
		2.5	90 ± 0.67	76 ± 2.52	2.09 ± 0.37	14 ± 0.58	8.0 ± 1.15	23.0 ± 0.33	5.26 ± 0.09
		5.0	97 ± 1.20	82 ± 1.20	2.73 ± 0.18	15 ± 1.15	11.0 ± 0.33	24.3 ± 0.00	5.71 ± 0.05
		7.5	82 ± 3.17	69 ± 2.33	2.18 ± 0.36	13 ± 1.20	8.3 ± 0.33	20.3 ± 1.20	4.84 ± 0.04
	75	0	69 ± 0.33	62 ± 4.16	1.61 ± 0.27	7 ± 0.58	5.3 ± 0.57	12.7 ± 0.33	4.23 ± 0.18
		2.5	78 ± 0.88	69 ± 0.67	1.30 ± 0.14	9 ± 1.20	7.0 ± 0.57	14.7 ± 0.67	4.82 ± 0.12
		5.0	85 ± 1.45	74 ± 0.33	2.23 ± 0.23	11 ± 0.33	10.0 ± 0.67	24.7 ± 0.67	5.22 ± 0.14
		7.5	86 ± 1.20	76 ± 1.52	1.85 ± 0.10	10 ± 1.20	8.0 ± 0.33	16.7 ± 1.67	4.00 ± 0.12
	50	0	65 ± 2.89	60 ± 1.73	1.23 ± 0.04	5 ± 1.69	5.3 ± 1.00	12.3 ± 0.88	3.63 ± 0.22
		2.5	70 ± 6.56	64 ± 3.00	1.39 ± 0.02	6 ± 1.67+	6.7 ± 0.00	14.0 ± 0.00	4.01 ± 0.04
		5.0	77 ± 3.59	70 ± 0.88	1.68 ± 0.07	7 ± 1.53	7.7 ± 0.33	16.0 ± 1.33	4.55 ± 0.04
		7.5	70 ± 2.65	64 ± 2.12	1.37 ± 0.05	6 ± 1.15	7.0 ± 0.33	13.7 ± 0.58	3.65 ± 0.013
LSD_0.05_			2.86	2.17	0.33	0.38	0.35	1.07	0.21

Varieties	Drought stress (%)	Melatonin (mM)	Seed yield (kg/fed)	Biological yield (kg/fed)	Straw yield (kg/fed)	Oil yield kg/fed
Letwania-9	100	0	558 ± 22.91	1388 ± 81.55	830 ± 31.57	168.0 ± 1.84
		2.5	654 ± 15.28	1794 ± 80.95	1140 ± 42.65	214.5 ± 2.68
		5.0	906 ± 30.00	2489 ± 106.61	1582 ± 65.65	304.4 ± 3.45
		7.5	663 ± 46.31	1453 ± 88.65	789 ± 74.52	208.8 ± 3.85
	75	0	309 ± 13.48	911 ± 75.65	601 ± 54.65	90.2 ± 2.68
		2.5	407 ± 37.23	1334 ± 100.65	926 ± 32.52	127.0 ± 3.12
		5.0	677 ± 57.52	1830 ± 120.65	1152 ± 42.65	220.7 ± 3.42
		7.5	613 ± 42.98	1185 ± 81.45	572 ± 42.35	194.9 ± 4.35
	50	0	157 ± 12.17	764 ± 41.57	607 ± 51.35	45.5 ± 5.14
		2.5	227 ± 15.76	966 ± 64.52	738 ± 61.52	69.2 ± 5.34
		5.0	369 ± 29.29	1269 ± 81.81	900 ± 63.51	116.2 ± 5.74
		7.5	316 ± 30.05	1101 ± 65.65	785 ± 65.85	98.0 ± 4.35
Sakha-2	100	0	395 ± 13.33	1388 ± 78.65	993 ± 62.35	131.9 ± 4.52
		2.5	494 ± 10.40	1794 ± 111.35	1300 ± 74.32	167.5 ± 4.35
		5.0	738 ± 34.17	2489 ± 110.65	1750 ± 62.35	251.7 ± 4.62
		7.5	509 ± 37.65	1453 ± 75.65	943 ± 42.32	169.0 ± 4.35
	75	0	405 ± 16.34	1268 ± 84.65	862 ± 57.65	126.4 ± 4.85
		2.5	573 ± 15.34	2853 ± 106.84	2279 ± 100.51	185.7 ± 6.74
		5.0	825 ± 60.45	1472 ± 76.85	647 ± 34.52	264.0 ± 6.75
		7.5	731 ± 16.77	1148 ± 81.65	416 ± 39.65	234.7 ± 6.48
	50	0	116 ± 10.11	1358 ± 74.68	1241 ± 38.65	37.2 ± 2.62
		2.5	253 ± 18.24	2500 ± 103.65	2246 ± 57.65	84.5 ± 3.42
		5.0	473 ± 17.08	2650 ± 120.35	2176 ± 67.85	153.3 ± 4.62
		7.5	338 ± 14.49	3511 ± 143.65	3172 ± 103.25	108.5 ± 4.65
LSD_0.05_			25.07	105.70	82.50	12.65

Each value represents the mean of three replicates ± SE

## Discussion

One of environmental stresses responsible for decrease in plant growth and productivity is drought stress. In this investigation, growth parameters were significantly decreased in the two varieties (Letwania-9 and Sakha-2) of flax plant under drought (decreasing WIR) as in Table 2. In harmony with our results of drought stress, Dawood and Sadak (2014), Sadak (2016a), Elewa et al. (2017) and Ezzo et al. (2018) stated that different growth criteria of canola, wheat, quinoa, and moringa plants decreased with drought stress and they referred these decreases to disorders induced by drought and generation of reactive oxygen species (ROS). These decreases in plant height might be due to decreases in cell elongation, cell turgor, cell volume and eventually cell growth (Banon et al. 2006). Moreover, drought affects plant–water relations, decreases shoot water contents, causes osmotic stress, inhibits cell expansion and cell division as well as growth of plants as a whole (Alam et al. 2014). Earlier studies have confirmed the promotive role of melatonin on growth of plant under stress viz, Arnao and Hernández-Ruiz (2014), Li et al. (2015), Liu et al. (2015), Ye et al. (2016), Cui et al. (2017), Kabiri, et al. (2018) and Debnath et al. (2019),  which referred this effect to the action of melatonin as a growth regulator and thus it could improve growth of various plants and as a protector against abiotic stress (Li et al. 2012). In addition, melatonin can act as a potential modulator of plant growth and development in a dose-dependent manner (Gao et al. 2018).

Photosynthesis is the physico-chemical process which use light energy to drive the biosynthesis of different organic compounds and consequently plant production (Ye et al. 2016). Drought reduced photosynthetic pigments in the two studied varieties of flax plant (Fig. 1). These obtained data are congruent with those obtained earlier on canola (Dawood and Sadak 2014), fenugreek Sadak (2016b), quinoa (Elewa et al. 2017) and Moldavian balm (Kabiri et al. 2018; Ezzo et al. 2018). These decreases might have resulted from photo-oxidation of pigments that cause oxidative, photosynthetic system damaging which leads to reduction in photosynthetic carbon assimilation (Din et al. 2011; Pandey et al. 2012). Moreover, the principle reason for decreasing photosynthetic rate is that, limitation of surrounding CO_2_ diffusion to the site of carboxylation, induced by stomatal closure resulted from water stress (Liu et al. 2013). Melatonin promotive effect on photosynthetic efficiency of flax plant are in agreement with those of Liu et al. (2015) on tomato, Ye et al. (2016) on maize, Cui et al. (2017) and Ke et al. (2018) on wheat and Kabiri et al. (2018) on Moldavian balm plants. This indicated that melatonin treatment improved the ultra-structure of chloroplasts under drought stress. In addition, melatonin treatment played an important role in preservation of chlorophyll and promotion of photosynthesis due to the antioxidant enzyme activities (as in Fig. 3a–d) and antioxidant contents and thus, inhibiting production of reactive oxygen species (Ezzo et al. 2018). Many authors referred the promotive effect of melatonin to the interactive effect of melatonin and other plant growth promoters such as kinetin and ABA on leaf senescence (Arnao and Hernandez-Ruiz 2017).

Under various environmental stresses such as drought stress, plants have developed different physiological and biochemical mechanisms to adapt or to tolerate stress. Figure 2 shows that drought stress and/or melatonin treatments enhanced phenolic content accumulation. Similar results were obtained under abiotic stress on different plant species, El-Awadi et al. (2017a) and Ezzo et al. (2018). These increases might be due to the effect of drought stress in induction of various metabolic processes disturbances which leads to increase in the synthesis of phenolic compounds (Keutgen and Pawelzik 2009). Actually, different abiotic stress as drought induced reactive oxygen species (ROS) accretion and this is generally coupled with changes in net carbon gain which may strongly affect the biosynthesis of carbon-based secondary compounds, particularly leaf polyphenols (Radi et al. 2013). Moreover, the promotive role of melatonin could result from its signaling function, via the induction of different metabolic processes and stimulate production of various substances, preferably operating under stress (Tan et al. 2012). Moreover, the enhancing role of melatonin on phenolic contents resulted from the induction of various metabolic pathway and promote the formation of different compounds especially operating under stress (Tan et al. 2012).

The accumulation of soluble carbohydrates in plants has been widely reported as a response to drought stress despite a significant decrease in net CO_2_ assimilation rate. The increased levels of TSS in response to different abiotic stress are confirmed earlier by Elewa et al. (2017) on quinoa plant and Ezzo et al. (2018) on moringa plant. Soluble carbohydrates could act as scavengers of ROS and contribute to increase in membrane stabilization. The increased levels of TSS might help in turgor maintenance and stabilization of cellular membrane (Hosseini et al. 2014). The results of the present work show that melatonin treatments decreased the harmful effect of drought stress on the two varieties of flax plants and increased their drought stress tolerance. Melatonin is a free radical scavenger and broad-spectrum antioxidant that might directly eliminate ROS when produced under stressful conditions.

In the present work, drought stress caused marked increases of proline and free amino acids, moreover, melatonin treatment caused increases in proline and free amino acids contents (Table 3). The osmotic adjustment in plants subjected to drought stress occurs by the accumulation of high concentrations of osmotically active compounds known as compatible solutes such as proline, glycinebetaine, soluble sugars, free amino acids and polyamines (Abd Elhamid et al. 2016). Earlier studies agreed with our obtained results Elewa et al. (2017) on quinoa plant and Ezzo et al. (2018) on moringa plant. They revealed that osmoprotectants (TSS, proline and free amino acids) play an important role in adaptation of cells to various adverse environmental conditions through raising osmotic pressure in cytoplasm, stabilizing proteins and membranes, and maintaining the relatively high water content obligatory for plant growth and cellular functions. Proline accumulation is considered as an indicator in several plant species under drought stress conditions, acting as an osmotic protectant and contributing to the turgor maintenance of cells (Elewa et al. 2017). Furthermore, the increases in proline content could be attributed to the decrease in proline oxidase activity under drought conditions (Bakry et al. 2012). Free amino acid accumulation associated with stress may actually be a part of an adaptive process contributing to osmotic adjustment (Sadak et al. 2010).

Drought stress caused significant increases in different enzymes of the two flax varieties (Fig. 3). These increases could be considered as an indicator of increased production of ROS and a build-up of a protective mechanism to reduce oxidative damage triggered by stress experienced by plants as mentioned by Abdelgawad et al. (2014), El-Awadi et al. (2017a), Kabiri et al. (2018) and Ezzo et al. (2018). Antioxidative enzymes are not part of this system but key elements in the defense mechanisms. Higher levels of enzyme activities in flax plant under water deficient may be due to its high resistance. NAD^+^ recovering and CO_2_ fixation at the Calvin cycle decrease under drought stress causing damage to cell membrane due to the increases of free radicals. Adverse environmental stresses increase catalase activity in several cycles of physiological processes. Stress conditions accompanied with higher content of ROS (especially H_2_O_2_) is detoxified by catalase (Dat et al. 2000). Superoxide dismutase (SOD) is the first defense enzyme that converts superoxide to H_2_O_2_, which can be scavenged by catalase (CAT) and different classes of peroxidases (POX). Shi et al. (2007) confirmed the essential role of antioxidant systems in plant tolerance of various environmental stress especially in tolerant cultivars that had higher activities of ROS-scavenging enzymes than susceptible ones. Melatonin and some of its metabolites are considered as endogenous free radical scavenger and antioxidants (Zhang et al. 2013) that could directly scavenge ROS such as H_2_O_2_ (Cui et al. 2017). Moreover, one of the main functions of melatonin, along with the activities of SOD and CAT may be to preserve intracellular H_2_O_2_ concentrations at steady –scale levels (Cui et al. 2017). Li et al. (2017) showed that melatonin, a potent long-distance signal, may be translocated from the treated leaves or roots of plant to distant untreated tissues via vascular bundles, leading to systemic induction of different abiotic tolerance. Moreover, studies on how melatonin interacts with stress signaling mechanisms have identified a complex relationship with ROS. Results concluded that, melatonin is a broad-spectrum direct antioxidant which can scavenge ROS with high efficiency. A detailed knowledge of melatonin chemistry and molecular interactions with ROS and with strong oxidants has been documented. As well as, melatonin treatments modulate the antioxidant enzymes by both up-regulating the transcript level and increasing the activity levels (Zhang et al. 2014a). Improving plant antioxidant systems has been considered the primary function of melatonin in plant stress tolerance (Zhang et al. 2015). Zhao et al. (2011) had proposed that melatonin protected *Rhodiola crenulata* cells against oxidative stress during cryo-preservation by increasing SOD and CAT activities.

Plant responses to water stress include growth parameters and biochemical changes that lead first to acclimation and later, as water stress become more severe leading to damage and the loss of plant parts (Chaves et al. 2003). Water stress reduced yield and yield components of flax varieties (Table 4a, b). Similar results were obtained by Dawood and Sadak (2014) on canola plant, Abd Elhamid et al. (2016) on fenugreek plant, Elewa et al. (2017) on quinoa plant. Water deficits affect plants in different ways, slowly developing water deficits decrease growth, by slowing rates of cell division and expansion due to loss of turgor (Lawlor and Cornic 2002) and/or resulted from osmotic effect of water stress which caused disturbances in water balance of stressed flax plant leading to decreases in photosynthetic pigments (Fig. 1) and consequently retarded growth rate (Table 2). In regards to melatonin effect, similar results were obtained by Li et al. (2012), Janas and Posmyk (2013), Zhang et al. (2014b) and Sadak (2016a, 2016b). The increases in growth characters caused by different melatonin concentrations might be due to the role of melatonin in alleviation growth inhibition, thus enabling plants to maintain a robust root system and improve photosynthetic capacity (Posmyk and Janas 2009) and thus increased yield and yield attributes.

## References

[CR1] Abd Elhamid EM, Sadak MS, Tawfik MM (2014). Alleviation of adverse effects of salt stress in wheat cultivars by foliar treatment with antioxidant 2—changes in some biochemical aspects, lipid peroxidation, antioxidant enzymes and amino acid contents. Agric Sci.

[CR2] Abd Elhamid EM, Sadak MS, Tawfik MM (2016). Physiological response of Fenugreek plant to the application of proline under different water regimes. Res J Pharm Biol Chem Sci.

[CR3] Abdelgawad ZA, Khalafaallah AA, Abdallah MM (2014). Impact of methyl jasmonate on antioxidant activity and some biochemical aspects of maize plant grown under Water Stress condition. Agric Sci.

[CR4] Alam MDM, Nahar K, Hasanuzzaman M, Fujita M (2014). Trehalose-induced drought stress tolerance: a comparative study among different Brassica species. POJ.

[CR5] Alam MN, Wang Y, Chan Z (2018). Physiological and biochemical analyses reveal drought tolerance in cool-season tall fescue (*Festuca arundinacea*) turf grass with the application of melatonin. Crop Pasture Sci.

[CR6] Allen GR, Jensen E, Wright James L, Burman Robert D (1989). Operational estimates of reference evapotranspiration. Agron J.

[CR7] AOAC (1990). Official methods of analysis.

[CR8] Arnao MB, Hernández-Ruiz J (2014). Melatonin: plant growth regulator and/or biostimulator during stress?. Trends Plant Sci.

[CR9] Arnao MB, Hernández-Ruiz J (2015). Functions of melatonin in plants: a review. J Pineal Res.

[CR10] Arnao MB, Hernández-Ruiz J (2017). Melatonin in its relationship to plant hormones. Ann Bot.

[CR11] Bakry BA, El-Hariri DM, Sadak MS, El-Bassiouny HMS (2012). Drought stress mitigation by foliar application of salicylic acid in two linseed varieties grown under newly reclaimed sandy soil. J Appl Sci Res.

[CR12] Bakry BA, Sadak MS, Moamen HT, Abd El Lateef EM (2013). Influence of humic acid and organic fertilizer on growth, chemical constituents, yield and quality of two flax seed cultivars grown under newly reclaimed sandy soils. Int J Acad Res.

[CR13] Banon SJ, Ochoa J, Franco JA, Alarcon JJ, Sanchez-Blanco MJ (2006). Hardening of oleander seedlings by deficit irrigation and low air humidity. Environ Exp Bot.

[CR14] Bates LS, Waldan RP, Teare LD (1973). Rapid determination of free proline under water stress studies. Plant Soil.

[CR15] Battipaglia G, De Micco V, Brand WA, Saurer M, Aronne G, Linke P, Cherubini P (2014). Drought impact on water use efficiency and intra-annual density fluctuations in *Erica arborea* on Elba (Italy). Plant Cell Environ.

[CR16] Bergmeyer HU (1974). Methods of enzymatic analysis I.

[CR17] Blum A (2017). Osmotic adjustment is a prime drought stress adaptive engine in support of plant production. Plant Cell Environ.

[CR18] Chapman HO, Pratt BE (1978). Methods of analysis for soils, plants and water.

[CR19] Chaves MM, Maroco JP, Pereira JS (2003). Understanding plant responses to drought from genes to the whole plant. Funct Plant Biol.

[CR20] Chen Y, Cao XD, Lu Y, Wang XR (2000). Effect of rare earth metal ions and their EDTA complexes on antioxidant enzymes of fish liver. Bull Environ Contam Toxicol.

[CR21] Chen J, Li Y, Luo Y, Tu W, Wan T (2019). Drought differently affects growth properties, leaf ultrastructure, nitrogen absorption and metabolism of two dominant species of Hippophae in Tibet Plateau. Acta Physiol Plant.

[CR22] Cui G, Zhao X, Liu S, Sun F, Zhang C, Xi Y (2017). Beneficial effects of melatonin in overcoming drought stress in wheat seedlings. Plant Physiol Biochem.

[CR23] Danil AD, George CM (1972). Peach seed dormancy in relation to endogenous inhibitors and applied growth substances. J Am Soc Hortic Sci.

[CR24] Darja KA, Trdan S (2008). Influence of row spacing on the yield of two flax cultivars (*Linum usitatissimum* L.). Acta Agriculturae Slovenica.

[CR25] Dat J, Vandenabeele S, Vranova EVMM, Van Montagu M, Inzé D, Van Breusegem F (2000). Dual action of the active oxygen species during plant stress responses. Cell Mol Life Sci CMLS.

[CR26] Dawood MG, Sadak MS (2014). Physiological role of glycinebetaine in alleviating the deleterious effects of drought stress on canola plants (*Brassica napus* L.). Middle East J Agric Res.

[CR27] Debnath B, Islam W, Li M, Sun Y, Lu X, Mitra S, Qiu D (2019). Melatonin mediates enhancement of stress tolerance in plants. Int J Mol Sci.

[CR28] Dhindsa R, Plumb-Dhindsa P, Thorpe T (1981). Leaf senescence correlated permeability, lipid peroxidation and decreased levels of superoxide dismutase and catalase. J Exp Bot.

[CR29] Din J, Kans SU, Ali J, Gurmani AR (2011). Physiological and agronomic response of canola varieties to drought stress. J Anim Plant Sci.

[CR30] El-Awadi ME, Sadak MS, Dawood MG, Khater MA, Elashtokhy MMA (2017). Amelioration the adverse effects of salinity stress by using *γ*-radiation in faba bean plants. Bull NRC.

[CR31] Elewa TA, Sadak MS, Saad AM (2017). Proline treatment improves physiological responses in quinoa plants under drought stress. Biosci Res.

[CR32] Ezzo MI, Abd Elhamid EM, Sadak MS, Abdalla AM (2018). Improving drought tolerance of Moringa plants by using trehalose foliar treatments. Biosci Res.

[CR33] Fleta-Soriano E, Díaz L, Bonet E, Munné-Bosch S (2017). Melatonin may exert a protective role against drought stress in maize. J Agron Crop Sci.

[CR34] Gao W, Zhang Y, Feng Z, Bai Q, He J, Wang Y (2018). Effects of melatonin on antioxidant capacity in naked oat seedlings under drought stress. Molecules.

[CR35] Gomez KA, Gomez AA (1984). Statistical producers for agriculture research.

[CR36] Hardeland R (2015). Melatonin in plants and other phototrophs: advances and gaps concerning the diversity of functions. J Exp Bot.

[CR37] He CY, Zhang GY, Zhang JG (2016). Physiological, biochemical and proteome profiling reveals key pathways underlying the drought stress responses of *Hippophae rhamnoides*. Proteomics.

[CR38] Homme PM, Gonzalez B, Billard J (1992). Carbohydrate content, frutane and sucrose enzyme activities in roots, stubble and leaves of rye grass (*Lolium perenne* L.) as affected by sources/link modification after cutting. J Plant Physiol.

[CR39] Hosseini SM, Hasanloo T, Mohammadi S (2014). Physiological characteristics, antioxidant enzyme activities, and gene expression in 2 spring canola (*Brassica napus* L.) cultivars under drought stress conditions. Turk J Agric For.

[CR40] Janas KM, Posmyk MM (2013). Melatonin, an underestimated natural substance with great potential for agricultural application. Acta Physiol Plant.

[CR41] Kabiri R, Hatami A, Oloumi H, Naghizadeh M, Nasibi F, Tahmasebi Z (2018). Foliar application of melatonin induces tolerance to drought stress in Moldavian balm plants (*Dracocephalum moldavica*) through regulating the antioxidant system. Folia Hortic.

[CR42] Kar M, Mishra D (1976). Catalase, peroxidase and polyphenoloxidase activities during rice leaf senescence. Plant Physiol.

[CR43] Ke Q, Ye J, Wang B, Ren J, Yin L, Deng X, Wang S (2018). Melatonin mitigates salt stress in wheat seedlings by modulating polyamine metabolism. Front Plant Sci.

[CR44] Keller J, Karmeli D (1975). Trickle design. Rain bird sprinkler.

[CR45] Keutgen AJ, Pawelzik E (2009). Impacts of NaCl stress on plant growth and mineral nutrient assimilation in two cultivars of strawberry. Environ Exp Bot.

[CR46] Kong FX, Hu W, Chao WL, Sang WL, Wang LS (1999). Physiological responses of the lichen *Xanthoparmelia mexicana* to oxidative stress of SO_2_. Environ Exp Bot.

[CR47] Lawlor DW, Cornic G (2002). Photosynthetic carbon assimilation and associated metabolism in relation to water deficits in higher plants. Plant Cell Environ.

[CR48] Li C, Wang P, Wei Z, Liang D, Liu C, Yin L, Jia D, Fu M, Ma F (2012). The mitigation effects of exogenous melatonin on salinity-induced stress in Malus hupehensis. J Pineal Res.

[CR49] Li C, Tan DX, Liang D, Chang C, Jia DE, Ma FM (2015). Melatonin mediates the regulation of ABA metabolism, free-radical scavenging and Stomatal behavior in two Malus species under drought stress. J Exp Bot.

[CR50] Li X, Tan DX, Jiang D, Liu F (2016). Melatonin enhances cold tolerance in drought-primed wild-type and abscisic acid-deficient mutant barley. J Pineal Res.

[CR51] Li X, Yu B, Cui Y, Yin Y (2017). Melatonin application confers enhanced salt tolerance by regulating Na^+^ and Cl^−^ accumulation in rice. Plant Growth Regul.

[CR52] Li X, Brestic M, Tan DX, Zivcak M, Zhu X, Liu S, Song F, Reiter RJ, Liu F (2018). Melatonin alleviates low PS I-limited carbon assimilation under elevated CO2 and enhances the cold tolerance of offspring in chlorophyll b-deficient mutant wheat. J Pineal Res.

[CR53] Li J, Liu J, Zhu T, Zhao C, Li L, Chen M (2019). The role of melatonin in salt stress responses. Int J Mol Sci.

[CR54] Lichtenthaler HK, Buschmann C, Wrolstad RE, Acree TE, An H, Decker EA, Penner MH, Reid DS, Schwartz SJ, Shoemaker CF, Sporns P (2001). Chlorophylls and carotenoids: measurement and characterization by UV–VIS spectroscopy. Current protocols in food analytical chemistry.

[CR55] Liu D, Wu L, Naeem MS, Liu H, Deng X, Xu L, Zhang F, Zhou W (2013). 5-Aminolevulinic acid enhances photosynthetic gas exchange, chlorophyll fluorescence and antioxidant system in oilseed rape under drought stress. Acta Physiol Plant.

[CR56] Liu J, Wang W, Wang L, Sun Y (2015). Exogenous melatonin improves seedling health index and drought tolerance in tomato. Plant Growth Regul.

[CR57] Maksup S, Roytrakul S, Supaibulwatana K (2014). Physiological and comparative proteomic analyses of Thai jasmine rice and two check cultivars in response to drought stress. J Plant Interact.

[CR58] Mukherjee SP, Choudhuri MA (1983). Implication of water stress -induced changes in the levels of endogenous ascorbic acid and hydrogen peroxide in Vigna seedling. Physiol Plant.

[CR59] Pandey HC, Baig MJ, Bhatt RK (2012). Effect of moisture stress on chlorophyll accumulation and nitrate reductase activity at vegetative and flowering stage in Avena species. Agric Sci Res J.

[CR60] Petit JR, Jouzel J, Raynaud D, Barkov NI, Barnola JM, Basile I, Bender M, Chappellaz J, Davis M, Delaygue M, Delmotte M, Kotiyakov VM, Legrand M, Stievenard M (1999). Climate and atmospheric history of the past 420,000 years from the Vostok ice core, Antarctica. Nature.

[CR61] Posmyk MM, Janas KM (2009). Melatonin in plants. Acta Physiol Plant.

[CR62] Radi AA, Farghaly FA, Hamada AM (2013). Physiological and biochemical responses of salt-tolerant and salt sensitive wheat and bean cultivars to salinity. J Biol Earth Sci.

[CR63] Rice-Evans CA, Miller NJ, Paganga G (1997). Antioxidant properties of phenolic compounds. Trends Plant Sci.

[CR64] Sadak MS (2016). Mitigation of salinity adverse effects on wheat by grain priming with melatonin. Int J ChemTech Res.

[CR65] Sadak MS (2016). Mitigation of drought stress on fenugreek plant by foliar application of trehalose. Int J ChemTech Res.

[CR66] Sadak MS, Abdelhamid MT, El-Saady AM (2010). Physiological responses of faba bean plant to ascorbic acid grown under salinity stress. Egypt J Agron.

[CR67] Shi Q, Ding F, Wang X, Wei M (2007). Exogenous nitric oxides protect cucumber roots against oxidative stress induced by salt stress. Plant Physiol Biochem.

[CR68] Snedecor GW, Cochran WG (1980). Statistical methods.

[CR69] Sun L, Li X, Wang Z, Sun Z, Zhu X, Liu S, Song F, Liu F, Wang Y (2018). Cold priming induced tolerance to subsequent low temperature stress is enhanced by melatonin application during recovery in wheat. Molecules.

[CR70] Tan DX, Hardeland R, Manchester LC, Korkmaz A, Ma S, Rosales- Corral S, Reiter RJ (2012). Functional roles of melatonin in plants, and perspectives in nutritional and agricultural science. J Exp Bot.

[CR71] Vartanian N, Hervochon P, Marcotte L, Larher F (1992). Proline accumulation during drought rhizogenesis in *Brassica napus* var. Oleifera. Plant Physiol.

[CR72] Wei W, Li QT, Chu YN, Reiter RJ, Yu XM, Zhu DH, Zhang WK, Ma B, Lin Q, Zhang JS (2015). Melatonin enhances plant growth and abiotic stress tolerance in soybean plants. J Exp Bot.

[CR73] Ye J, Wang S, Deng X, Yin L, Xiong B, Wang X (2016). Melatonin increased maize (*Zea mays* L.) seedling drought tolerance by alleviating drought-induced photosynthetic inhibition and oxidative damage. Acta Physiol Plant.

[CR74] Yemm EW, Cocking EC (1955). The determination of amino acids with ninhydrin. Analyst.

[CR75] Yemm EW, Willis AJ (1954). The respiration of barley plants. IX. The metabolism of roots during assimilation of nitrogen. New Phytotol.

[CR76] Zhang HM, Zhang YQ (2014). Melatonin: a well-documented antioxidant with conditional pro-oxidant actions. J Pineal Res.

[CR77] Zhang N, Zhao B, Zhang HJ, Weeda S, Yang C, Yang ZC, Ren S, Guo YD (2013). Melatonin promotes water-stress tolerance, lateral root formation, and seed germination in cucumber (*Cucumis sativus* L.). J Pineal Res.

[CR78] Zhang HJ, Zhang N, Yang RC, Wang L, Sun QQ, Li DB, Cao YY, Weeda S, Zhao B, Ren S, Guo YD (2014). Melatonin promotes seed germination under high salinity by regulating antioxidant systems, ABA and GA4 interaction in cucumber (*Cucumis sativus* L.). J Pineal Res.

[CR79] Zhang N, Sun Q, Zhang H, Cao Y, Weeda S, Ren S, Guo Y (2014). Roles of melatonin in abiotic stress resistance in plants. J Exp Bot.

[CR80] Zhang N, Sun Q, Zhang H, Cao Y, Weeda S, Ren S, Guo YD (2015). Roles of melatonin in abiotic stress resistance in plants. J Exp Bot.

[CR81] Zhao Y, Qi LW, Wang WM (2011). Melatonin improves the survival of cryopreserved callus of *Rhodiola crenulata*. J Pineal Res.

[CR82] Zuo Z, Sun L, Wang T, Miao P, Zhu X, Liu S, Song F, Mao H, Li X (2017). Melatonin improves the photosynthetic carbon assimilation and antioxidant capacity in wheat exposed to nano-ZnO stress. Molecules.

